# ^18^F-sodium fluoride PET-CT visualizes both axial and peripheral new bone formation in psoriatic arthritis patients

**DOI:** 10.1007/s00259-022-06035-w

**Published:** 2022-11-12

**Authors:** Jerney de Jongh, Robert Hemke, Gerben J. C. Zwezerijnen, Maqsood Yaqub, Irene E. van der Horst-Bruinsma, Marleen G. H. van de Sande, Arno W. R. van Kuijk, Alexandre E. Voskuyl, Conny J. van der Laken

**Affiliations:** 1 Department of Rheumatology and Clinical Immunology, Amsterdam UMC, Location VUmc, P.O. Box 7057, Amsterdam, the Netherlands; 2 Department of Radiology & Nuclear Medicine, Amsterdam UMC, Location AMC, 1007 MB Amsterdam, The Netherlands; 3 Department of Radiology & Nuclear Medicine, Amsterdam UMC, Location VUmc, Amsterdam, The Netherlands; 4 Department of Rheumatology, Radboud University Medical Center, Nijmegen, The Netherlands; 5 Department of Rheumatology and Clinical Immunology, Amsterdam UMC, Location AMC, Amsterdam, the Netherlands; 6 Department of Rheumatology, Reade, Amsterdam, the Netherlands

**Keywords:** ^18^F-sodium fluoride PET/CT, Psoriatic arthritis, Feasibility, Bone formation

## Abstract

**Purpose:**

As bone formation is associated with psoriatic arthritis (PsA), positron emission tomography (PET) using a ^18^F-Fluoride tracer may enable sensitive detection of disease activity. Our primary aim was to determine the feasibility of whole-body ^18^F-sodium fluoride PET-CT in clinically active PsA patients to depict new bone formation (as a reflection of disease activity) at peripheral joints and entheses. Our secondary aim was to describe ^18^F-sodium fluoride findings in the axial skeleton.

**Methods:**

Sixteen patients (female 10/16, age 50.6 ± 8.9 years) with PsA fulfilling CASPAR criteria or with a clinical diagnosis of PsA according to the treating rheumatologist and with ≥ 1 clinically active enthesitis site were included. Of each patient, a whole-body ^18^F-sodium fluoride PET-CT scan was performed. All scans were scored for PET-positive lesions at peripheral joints, enthesis sites and the spine. Clinical disease activity was assessed by swollen/tender joint count 44, enthesitis according to MASES and SPARCC scores.

**Results:**

Out of 1088 evaluated joints, 109 joints showed PET enhancement, mainly in the interphalangeal and metatarsal joints of the feet (14/109, 12.9%) and the distal interphalangeal joints of the hands (14/109, 12.9%). PET positivity was found at 44/464 enthesis sites, mainly at the patella tendon insertion (11/44, 25%) and quadriceps tendon insertion (10/44, 22.7%). Of the PET-positive joints and enthesis sites, respectively 18.2% and 29.5% were clinically positive; 81.8% and 70.5% of the PET-positive joints and entheses respectively were clinically asymptomatic. In 11 patients, ≥ 1 axial PET-positive lesion was observed, mainly in the cervical spine.

**Conclusions:**

New molecular bone formation was observed on ^18^F-sodium fluoride PET-CT scans, in all domains in which PsA disease activity can be observed, with a substantial part showing no clinical symptoms.

**Clinical trial registration:**

EudraCT: 2017-004,850-40, registered on 13 December 2017.

**Supplementary Information:**

The online version contains supplementary material available at 10.1007/s00259-022-06035-w.

## Introduction

Psoriatic arthritis (PsA) is a chronic, inflammatory, musculoskeletal disease associated with psoriasis [1] and several musculoskeletal manifestations, including arthritis, dactylitis, spondylitis and enthesitis. The last of these is a key pathophysiological feature that negatively affects the quality of life [2–6]. Previous data indicate that enthesitis underlies a variety of manifestations of PsA [7]. A synovial-entheseal complex has been described, pointing at a close relationship between an enthesis and the synovial membrane, suggesting that entheseal abnormalities might trigger secondary joint synovitis [8]. In addition, enthesitis plays a relevant role in dactylitis and axial disease activity as well [9]. Detection of enthesitis may therefore enable early assessment of PsA disease activity, which in turn may lead to the start of early treatment that can potentially improve all disease manifestations of PsA [8, 9].

Clinical assessment of enthesitis is challenging and has limited accuracy, as it is based only on the presence of tenderness and general soft-tissue swelling [6]. Moreover, clinical assessment is often unable to identify bursitis, erosions or calcifications [10, 11]. Advanced imaging techniques such as ultrasound (US) and magnetic resonance imaging (MRI) have shown promise to sensitively detect entheseal inflammation [12–14]. However, limitations of these techniques have also been described. In US, bodyweight and repetitive physical activity or overloading can influence structural entheseal lesions and can thus bias the observation of disease-related enthesitis [15–17]. In addition, this technique cannot be used to detect axial and more deeply located enthesitis. As for MRI, conventional MRI is limited to the selected field of view [18]. Whole-body MRI (WBMRI) may be an alternative, but the image quality and reproducibility of distal peripheral sites are low [19]. Moreover, the slice thickness (5–6 mm) causes a lower readability of some entheses, such as at the costochondral joints [20, 21]. Structural entheseal lesions, which are characterized by periosteal proliferation and new bone formation, have been identified in psoriasis patients without evidence of PsA using high-resolution peripheral quantitative computed tomography (HR-pQCT) and are described as an independent marker for later PsA development [22, 23]. However, HR-pQCT is mainly suitable for small body parts due to its limited field of view.

Positron emission tomography (PET) may be a promising alternative for detection of disease activity in the whole body since it combines picomolar depiction of pathologic processes with anatomical low-dose computed tomography (CT) imaging as a reference [24, 25]. By application of specific tracers, molecular targets of interest can be visualized. One such tracer is ^18^F-sodium fluoride (^18^F-NaF), which depicts new bone formation as a consequence of osteoblastic activity. New bone formation is an important hallmark of spondyloarthritis [26]. We and others have recently demonstrated that ^18^F-NaF PET allows for sensitive and specific imaging of new bone formation in ankylosing spondylitis (AS) patients [27, 28]. Since enthesitis activity in PsA can be accompanied by new bone formation (e.g. peripheral formation in osteophytes and axial formation in syndesmophytes), ^18^F-NaF PET may enable sensitive detection of skeletal disease manifestations in PsA.

Therefore, the primary aim of this study was to determine the feasibility of whole-body ^18^F-NaF PET-CT in clinically active PsA patients to depict new bone formation (as reflection of disease activity) at peripheral joints and entheses. Secondly, we aimed to describe ^18^F-NaF findings in the axial skeleton of clinically active PsA patients.

## Material and methods

### Patients and clinical assessment

Consecutive PsA patients were included between October 2018 and December 2020 in this prospective study. Patients visited the outpatient clinic of a tertiary rheumatology centre (Amsterdam UMC, locations VUmc and AMC, and Reade). Patients (≥ 18 years) were included if they fulfilled the Classification criteria for Psoriatic arthritis (CASPAR) [4] or had a clinical diagnosis of PsA according to the treating rheumatologist, had an enthesitis score of ≥ 1 according to the Maastricht Ankylosing Spondylitis Enthesitis Score (MASES) (range 0–13) [29] and/or the Spondyloarthritis Research Consortium of Canada (SPARCC) enthesitis index (range 0–16) [30] and had a clinical indication to start biological therapy. Exclusion criteria were the use of an experimental drug in the previous 3 months, pregnancy or breastfeeding. It was allowed to continue the use of cDMARDS and NSAIDs, given that the dosage was stable for ≥ 3 months prior to inclusion. After inclusion, clinical and demographical data were collected. Clinical disease activity was assessed, including swollen joint count (SJC)/tender joint count (TJC) 44, MASES, SPARCC, inflammatory back pain (IBP) (yes/no) assessed by treating physician or researcher [31], dactylitis (yes/no), Patient Global Disease Activity (PGDA) score (range 0–10), erythrocyte sedimentation rate (ESR) and C-reactive protein (CRP).

The Medical Ethics Review Committee of the VU University Medical Center approved the study protocol. All patients gave written informed consent prior to participation in the study.

### ^18^F-sodium fluoride PET scanning

PET-CT scans were performed, using either Ingenuity TF, Vereos (Philips Healthcare, Andover, MA, USA) or Biograph mCT Flow VG70A (Siemens Healthineers, Erlangen, Germany) PET-CT scanners. An 18-gauge needle infusion line was inserted in the antecubital vein in both arms, one for the withdrawal of blood and one for the tracer injection. A radioactivity dose of 102.8 ± 4.5 MBq ^18^F-NaF was injected, followed by a catheter flush with 20 mL NaCl 0.9%. To accurately determine the amount of injected radioactivity, residual activity was measured. Patients were scanned in supine position, with their hands placed on their lap. In order to limit hand movement and misregistration, patients placed their hands in a vacuum bag that was placed on their lap.

A whole-body (3 min per field of view (FOV)) PET scan was performed, starting 45 min after tracer injection, covering the skull base to the mid-thigh (with hands in the FOV), knees and ankles/feet. This scan was preceded by a 30-mAs low-dose CT scan.

PET data were normalized and corrected for attenuation, decay and scatter, using previously described procedures [32]. All scans were reconstructed as 144 × 144 matrices with a pixel size of 4 × 4 × 4 mm. The dynamic scans were reconstructed into 22 frames with progressively increasing frame durations (1 × 10, 4 × 5, 2 × 10, 2 × 20, 4 × 30, 4 × 60, 1 × 150 and 4 × 300 s). Images were transferred to offline workstations for visual analysis.

### Imaging analysis

The static PET-CT scans have been independently assessed for PET-positive lesions, by a board-certified musculoskeletal radiologist (R.H.) and a board-certified nuclear medicine physician (G.Z.) who were blinded for the clinical data. In case of disagreement, an adjudication read by a third reader (C.v.d.L.) together with the nuclear medicine physician (G.Z.) has been performed in order to reach a definitive score. Visual analysis was performed using standard 3D image viewing software, using the low-dose CT scan for anatomical reference. In view of the proof-of-concept design of this study, all foci of increased ^18^F-NaF uptake at peripheral joints and entheses and in the axial skeleton were described. Images were dichotomously scored for tracer uptake (positive or negative), using local background as a reference.

PET positivity was assessed at the following joints: temporomandibular joints, sternoclavicular joints, acromioclavicular joints, shoulders, elbows, wrists, metacarpophalangeal (MCP) 1–5 joints, proximal interphalangeal (PIP) 1–5 joints, distal interphalangeal (DIP) 2–5 joints, hips, knees, ankles, midtarsal joints, metatarsophalangeal (MTP) 1–5 joints and IP 1–5 joints of the feet. A total of 1088 joints were assessed (68 per patient). All enthesis locations as described in both the clinical MASES and SPARCC scores have been assessed for PET positivity. A total of 464 enthesis locations were assessed (29 per patient). Axial PET positivity was assessed at the following locations: processus spinosus, costovertebral joints, facet joints, anterior and posterior sides of vertebrae, superior and inferior endplates and the sacroiliac joints.

The low-dose CT scan was used for anatomical localization of the PET signals. In addition, it was applied to screen for major structural changes, in particular to identify major osteoarthritis lesions in the hand and feet joints and in the axial skeleton. ^18^F-NaF PET-positive lesions were classified as likely PsA related, in the absence of (major) structural abnormalities that were compatible with primary osteoarthritis as far as the low dose CT allowed such interpretation [33, 34].

### Statistical analysis

Statistical analysis were performed using SPSS version 28.0 for Windows. Continuous variables are summarized using mean (S.D.) or as median and interquartile range (IQR) in case the variables are not normally distributed. For comparative analysis between clinical findings and PET findings, only the 44 joints as described in the clinical SJC/TJC 44 score have been included.

## Results

### Patients

We included 16 clinically active PsA patients with a mean age of 50.6 (S.D. 8.9) years. Baseline characteristics and disease activity assessments are summarized in Table 1. ^18^F-NaF PET-CT scans were well tolerated by all patients. Images of both the axial and peripheral skeleton had a good quality for assessment of the presence of PET-positive lesions.

**Table 1:** Patient demographics and disease activity assessments. Results are shown as mean (S.D.) unless otherwise noted. CRP: C-reactive protein. ESR: erythrocyte sedimentation rate

Characteristic	Group (*n* = 16)
Females, no. (%)	10 (62.5)
Age, years, median (IQR)	50 (12.3)
Disease duration since diagnosis, years, median (IQR)	6.5 (6.8)
Fulfilling CASPAR criteria, no. (%)	13 (81.3)
Biological treatment naïve at time of inclusion, no. (%)	10 (62.5)
Enthesitis score according to MASES	3.9 (3.6)
Enthesitis score according SPARCC, median (IQR)	3.5 (3.8)
Patient global disease activity (0–10)	7.2 (1.3)
CRP, mg/L, median (IQR)	3.0 (4.0)
ESR, mm/h	12.8 (11.1)
Inflammatory back pain, no. (%)	4 (25.0)
44 swollen joint count, no	3.0 (3.0)
44 tender joint count, no	7.0 (8.0)
Dactylitis, no. (%)	2 (12.5)

### ^18^F-sodium fluoride PET findings

#### Peripheral joints

**Fig. 1:**
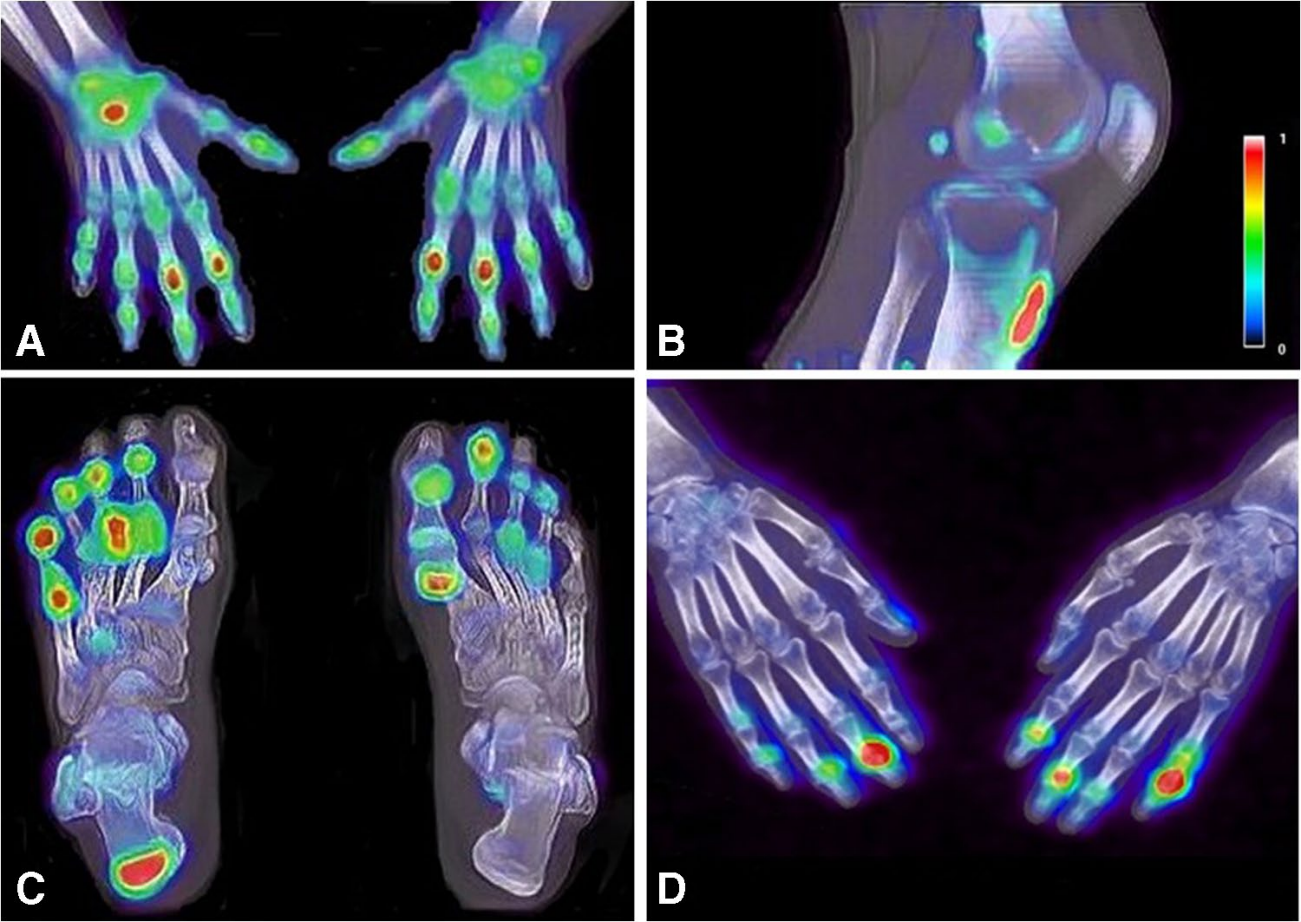
^18^F-NaF enhancement in the right wrist and proximal interphalangeal joints of the hands (**A**), at the patella tendon insertion (**B**), in the metatarsophalangeal and interphalangeal joints of the feet and the right Achilles tendon (**C**) and in the distal interphalangeal joints of the hands (**D**)

Thirteen out of 16 patients showed ^18^F-NaF uptake in one or more joints. In total, 109 out of 1088 evaluated joints were PET positive (Fig. 1A, C, D). PET enhancement was found in nearly all joints, except the shoulder, elbow and hip joints. A detailed overview of PET findings at the joint level is summarized in Table 2. Two out of 16 patients showed ^18^F-NaF uptake at a dactylitis site, both located in the feet.

**Table 2:** Overview of frequencies of visual PET-positive lesions per peripheral joint

Joint	Frequency of visual PET-positive, *n* (% of total)
Temporomandibular joint	1 (0.9)
Sternoclavicular joint	4 (3.7)
Acromioclavicular joint	8 (7.3)
Shoulders	0 (0)
Elbows	0 (0)
Wrists	2 (1.8)
MCP1 joints	1 (0.9)
MCP 2–5 joints	4 (3.7)
PIP joints hands	12 (11.0)
DIP joints hands	14 (12.9)
Hips	0 (0)
Knees	13 (11.9)
Ankles	9 (8.3)
Midtarsal joints	14 (12.8)
MTP 1 joints	8 (7.3)
MTP 2–5 joints	5 (4.6)
IP 1–5 foot	14 (12.9)
**Total**	**109 (100)**

#### Entheses

Fourteen out of 16 patients showed PET enhancement at one or more enthesis sites. In total, 44 out of 464 (9.5%) evaluated that enthesis sites were PET positive. The distal patella tendon insertion (11/44, 25.0%) (Fig. 1B) and the quadriceps tendon insertion (10/44, 22.7%) were the enthesis sites that most often showed PET positivity. Five out of 44 (11.4%) PET-positive lesions were located at the Achilles tendon (Fig. 1C). No PET-positive lesions were found at enthesis sites that were located in the pelvis (spina iliaca posterior superior (SIPS), spina iliaca anterior superior (SIAS) and crista iliaca). Furthermore, no PET enhancement was observed in both the 1st and 7th costochondral joints (Table 3).

**Table 3:** Overview of frequencies of visual PET-positive lesions per enthesis location

Enthesis location	Frequency of visual PET-positive, *n* (% of total)
Supraspinatus insertion	3 (6.8)
Lateral epicondyle humerus	5 (11.4)
Medial epicondyle humerus	3 (6.8)
Trochanter major	2 (4.5)
Quadriceps insertion	10 (22.7)
Patella tendon insertion	11 (25.0)
Achilles tendon insertion	5 (11.4)
Plantar fascia insertion	5 (11.4)
1st costochondral joint	0 (0)
7th costochondral joint	0 (0)
Spina iliaca posterior superior	0 (0)
Spina iliaca anterior posterior	0 (0)
Crista iliaca	0 (0)
Processus spinosus L5	0 (0)
**Total**	**44 (100)**

### Comparison PET-CT data with clinical data

The following joints/entheses have been included for the comparison of ^18^F-NaF PET-CT enhancement with clinical data: sternoclavicular joints, acromioclavicular joints, shoulders, elbows, wrists, MCP 1–5 joints, PIP 1–5 joints, knees, ankles and MTP 1–5 joints, all entheses that are included in MASES and SPARCC score (in correspondence with available clinical data of these sites). Twelve out of 66 (18.2%) PET-positive joints were also clinically positive (tender or swollen), leaving 81.8% of PET-positive joints that were clinically negative. Most PET-negative sites were also clinically negative (611/638, 95.8%). The (dis)agreement level of PET outcome in only tender, only swollen or tender and swollen joints was similar. Similar numbers were found for entheses, namely 13/44 (29.5%) PET-positive entheses were also clinically positive, and 70.5% of the PET-positive entheses were clinically negative. Detailed comparisons at the joint/enthesis level are summarized in respectively Supplementary Table 1 and 2. Two clinically active dactylitis sites also showed PET enhancement.

### ^18^F-sodium fluoride PET findings in the spine and sacroiliac joints

**Fig. 2:**
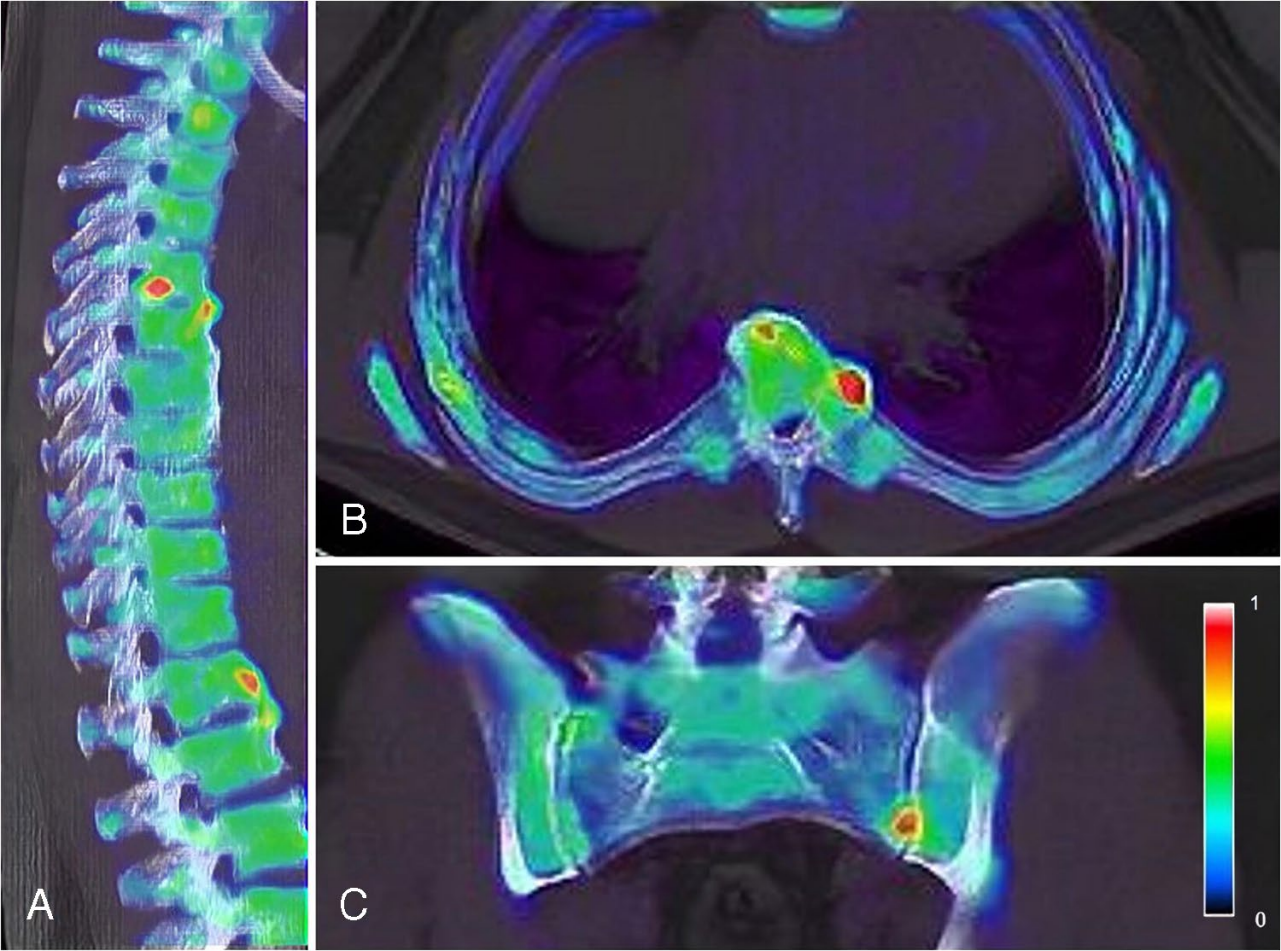
^18^F-NaF enhancement in the thoracic spine, at the anterior side of vertebrae and at a costovertebral joint (**A**, **B**) and in the left sacroiliac joint (**C**)

Eleven out of 16 patients showed ^18^F-NaF uptake at one or more sites at the axial level (Fig. 2A, B; Supplementary Table 3). A total of 51 PET-positive lesions were found in the spine, most frequently located in the cervical spine (19/51, 37.3%). Mainly the facet joints (11/19, 57.8%) were PET avid. In the thoracic and lumbar spine, a total of respectively 12 and 18 PET-positive lesions were found. In these segments of the spine, the anterior side of vertebrae (7/12, 58.3% and 5/18, 27.7% respectively) and facet joints (3/12, 25.0% and 9/18, 50.0% respectively) were most frequently PET positive. The posterior side of vertebrae was only found to be PET positive in the cervical spine (1/19, 5.3%), but not in the thoracic or lumbar spine. Two patients showed PET enhancement in one sacroiliac joint (Fig. 2C). Out of the 51 axial lesions, 11 (21.6%) lesions did not show major structural degenerative/osteoarthritis on low-dose CT and were suspected for PsA-related uptake (see examples in Fig. 2). In the four patients with IBP, a total of 7 PET-positive lesions were found, whereas the majority of axial lesions (*n* = 44) were detected in patients that did not report IBP. The patients that showed PET enhancement in the sacroiliac joints did not report IBP.

## Discussion

This is, to our knowledge, the first feasibility study on whole-body ^18^F-NaF PET-CT imaging in PsA patients. Our data demonstrate that ^18^F-NaF PET-CT can detect new bone formation, at sites of peripheral joints and various entheses and in dactylitis. In addition, several lesions with ^18^F-NaF uptake, suspect for PsA activity, could be demonstrated in the axial skeleton. Taken together, these findings suggest that ^18^F-NaF PET-CT may be a novel clinically valuable tool to detect whole-body disease activity of PsA reflected by new bone formation in all disease domains of PsA, depicted in one scan.

Several studies have aimed to visualize disease activity of PsA. An association has been described between enthesitis and extensive adjacent osteitis in both peripheral joints and the spine and is most likely the base for bone formation and related ^18^F-NaF PET tracer uptake [27, 35, 36]. Thus far, ^18^F-NaF PET-CT was only investigated previously in one other study of the DIP joints in a limited number of PsA patients, showing tracer uptake in the bone-enthesis-nail complex [37]. In our study, we have shown that ^18^F-NaF PET-CT can highly sensitively depict the activity of all PsA manifestations in one whole-body scan. In this perspective, the PET-CT tool also has advantages over currently applied US and MRI in PsA, as is outlined in “Introduction” [19, 20]. Moreover, the technique visualizes molecular new bone formation, possibly another aspect of the disease activity than US and MRI that primarily image inflammatory activity. Therefore, ^18^F-NaF PET-CT and MRI/US may be complementary in providing objective PsA disease activity assessment. In addition, although direct comparative studies between ^18^F-fluorodeoxyglucose (^18^F-FDG) and ^18^F-NaF in PsA are lacking, a comparative study by our group between ^18^F-FDG and ^18^F-NaF in AS patients revealed that disease activity on PET-CT is superiorly visualized by imaging new bone formation rather than inflammation [38]. As both PsA and AS are part of the spondyloarthropathy (SpA) spectrum, based on current studies, ^18^F-NaF seems to be the preferred PET tracer over ^18^F-FDG with regard to disease activity visualization of SpA. Whether new bone formation precedes, co-exists (dependently or independently) or follows inflammatory activity in PsA still needs to be unravelled [39]. Imaging studies with the different modalities and associated histological validation could support future pathogenetic research.

We observed a high number of clinically asymptomatic peripheral joint and entheseal lesions with ^18^F-NaF PET enhancement. These results are in line with those of Tan et al. who observed more PET enhancement in asymptomatic DIP joints in PsA patients as well, compared to healthy controls [37]. In fact, a high level of discrepancy between PET-CT and clinical findings may be expected, since ^18^F-NaF PET-CT visualizes molecular new bone formation and clinical assessment is directed at inflammatory activity. As stated above, the association of inflammation and new bone formation in PsA is not clear yet, and may occur (partly) independently and/or at different time points [40–43]. Several data suggest highly sensitive detection of subclinical disease activity by^18^F-NaF PET-CT that may precede clinical symptoms and/or radiological abnormalities/progression of PsA. Positive lesions in anterior corners of vertebrae on ^18^F-NaF PET-CT in spondyloarthritis patients have been found to be associated with local syndesmophyte formation 2 years later in time [28]. In addition, bone remodelling has already been demonstrated at the entheses in MCP joints in psoriasis patients without clinically diagnosed PsA (yet) [42]. Apart from depiction of another disease activity aspect by ^18^F-NaF PET-CT than clinical assessment, there are probably also other reasons for disagreement between the two. This is also reflected by reported low-moderate agreement between clinical and US and MRI imaging findings that primarily focus on inflammatory activity in joints and entheses [12, 20, 44–47]. Although clinical joint and enthesis counts are validated outcome measures, several limitations are known. Enthesitis is generally difficult to examine clinically in a reliable way, since this is only based on pain provoked by local pressure, and deeper located entheses cannot be assessed by clinical examination at all [6]. In addition, it is a general finding in clinical practice that certain joints, including those in midfoot and IP joints in toes, are difficult to assess for presence of disease activity. Especially the assessment of swollen joints often has a very poor inter-observer agreement [48, 49] while swollen joints in particular are associated with radiographic joint progression and are therefore crucial to include in disease activity assessments [50]. ^18^F-NaF uptake in asymptomatic peripheral sites may also be related to local degenerative changes, as these can be ^18^F-NaF positive as well [51, 52]. However, our analysis of the ^18^F-NaF PET-CT scans using the low-dose CT to screen for major degenerative/osteoarthritis changes revealed that the majority of peripheral joints with ^18^F-NaF tracer uptake did not show major osteoarthritis but partly showed typical PsA structural abnormalities including bone formation, erosions and pencil-in-cup deformation. In addition, potential bias of degenerative related ^18^F-NaF uptake in peripheral joints is less likely, as ^18^F-NaF-positive lesions were also observed in younger patients without any signs of local degeneration on low-dose CT. Moreover, in previous longitudinal ^18^F-NaF PET-CT data we collected in AS patients, we found that ^18^F-NaF uptake was responsive to anti-tumour necrosis factor (aTNF) treatment in typical AS spine lesions, which supports the potential of ^18^F-NaF PET-CT to image molecular new bone formation as part of spondyloarthritis disease activity [27]. Together, the current study data point at clinical and subclinical detection of PsA activity in bone by ^18^F-NaF PET-CT. The clinical relevance of asymptomatic PET lesions in PsA should be further addressed in longitudinal studies, relating PET outcome with clinical and radiological follow-up over time.

Axial involvement in PsA is associated with worse outcomes, but is an often underdiagnosed aspect of the disease [53–55]. In 20–25% of patients, subclinical axial involvement is present, without clinical features demonstrating that ^18^F-NaF PET-CT scans can depict lesions in the spine and SI joints in often clinically asymptomatic patients, indicating that this imaging technique visualizes signs of axial bone formation, even before clinical symptoms arise. An important differential diagnosis for this uptake, however, is local degeneration. Nevertheless, approximately 20% of our spinal lesions were identified as likely PsA related (lacking major primary degenerative changes on low-dose CT), although some misclassification cannot be ruled out since we used (non-diagnostic) low-dose CT for interpretation. These findings should be further explored in longitudinal studies. In fact, in our previously published ^18^F-NaF PET-CT study in ankylosing spondylitis patients, we found that in particular PET-positive costovertebral joints and SI joints were responsive to anti-TNF treatment and could distinguish between clinical responders and non-responders, pointing at detection of SpA-related lesions in the axial skeleton [27]. The lack of comparative radiological imaging with diagnostic anatomical modalities in our study precludes any in-depth recognition of disease-related uptake patterns versus degenerative-related uptake patterns. Nonetheless, our aim was to describe, as a first whole-body feasibility study, the ^18^F-NaF findings in the axial skeleton of clinically active PsA patients. Future research should focus on differentiation between typical PsA and typical degenerative lesions in order to exclude the degenerative lesions from analysis on PsA related disease, resulting in a comprehensible reflection of the extent of disease related bone formation in PsA patients.

Apart from the above-described lacking comparative diagnostic anatomical modalities, this feasibility study included some other limitations. Firstly, this study was performed in a small group of PsA patients and further validation of our results in larger cohorts is needed. Secondly, the study is limited by the lack of clinical information on the DIP, midtarsal and the IP joints of the feet. In these joints, PET positivity was frequently found; thus, the comparison of PET and clinical findings may have had a different outcome for these joints.

## Conclusion

^18^F-NaF PET-CT scans can visualize new bone formation, pointing at local PsA activity, at all peripheral disease activity sites and possibly in the axial skeleton. ^18^F-NaF PET-CT may add information to clinical disease activity assessment, reflected by a high number of clinically negative, PET-positive sites on top of concordant findings.

## Supplementary Information

Below is the link to the electronic supplementary material.

Supplementary file1 (DOCX 18 KB)

## Data Availability

The datasets generated and/or analysed during the current study are available from the corresponding author on reasonable request.
