# Supplementary material for: 18F-sodium fluoride PET-CT visualizes both axial and peripheral new bone formation in psoriatic arthritis patients
Source: Eur J Nucl Med Mol Imaging. 2022 Nov 12;50(3):756–64. doi: 10.1007/s00259-022-06035-w (PMC9852163; doi:10.1007/s00259-022-06035-w)
Supplement: Supplementary file 1 — Supplementary file1 (DOCX 18 KB) [file 259_2022_6035_MOESM1_ESM.docx]

**Supplementary information**

**Article title:** [^18^F]Fluoride PET-CT visualizes both axial and peripheral new bone formation in psoriatic arthritis

**Journal name:** European Journal of Nuclear Medicine and Molecular Imaging

**Author names:** J. de Jongh, R. Hemke, G.J.C. Zwezerijnen, M. Yaqub, I.E. van der Horst-Bruinsma, M.G.H. van de Sande, A.W.R. van Kuijk, A.E. Voskuyl, C.J. van der Laken

**Affiliation and e-mail address of the corresponding author:** Amsterdam UMC, Vrije Universiteit Amsterdam, Department of Rheumatology and Clinical Immunology, De Boelelaan 1117, Amsterdam, Netherlands, e-mail: [j.dejongh2@amsterdamumc.nl](mailto:j.dejongh2@amsterdamumc.nl)

Supplementary Table 1. Detailed comparison between PET enhancement and clinical data per peripheral joint

| **Joint** |  | **Visual PET-positive** | **Visual PET-negative** | **Total** |
| --- | --- | --- | --- | --- |
| Sternoclavicular joint | Tender or swollen | 0 | 0 | 0 |
|  | Non tender or swollen | 4 | 28 | 32 |
|  | Total | 4 | 28 | 32 |
|  | | | | |
| Acromioclavicular joint | Tender or swollen | 0 | 0 | 0 |
|  | Non tender or swollen | 8 | 24 | 32 |
|  | Total | 8 | 24 | 32 |
|  | | | | |
| Shoulders | Tender or swollen | 0 | 10 | 10 |
|  | Non tender or swollen | 0 | 22 | 22 |
|  | Total | 0 | 32 | 32 |
|  | | | | |
| Elbows | Tender or swollen | 0 | 4 | 4 |
|  | Non tender or swollen | 0 | 28 | 28 |
|  | Total | 0 | 32 | 32 |
|  | | | | |
| Wrists | Tender or swollen | 1 | 5 | 6 |
|  | Non tender or swollen | 1 | 25 | 26 |
|  | Total | 2 | 30 | 32 |
|  | | | | |
| MCP1 joints | Tender or swollen | 1 | 4 | 5 |
|  | Non tender or swollen | 0 | 27 | 27 |
|  | Total | 1 | 31 | 32 |
|  | | | | |
| MCP 2 – 5 joints | Tender or swollen | 1 | 15 | 16 |
|  | Non tender or swollen | 3 | 109 | 112 |
|  | Total | 4 | 124 | 128 |
|  | | | | |
| PIP joints | Tender or swollen | 4 | 14 | 18 |
|  | Non tender or swollen | 8 | 134 | 138 |
|  | Total | 12 | 148 | 160 |
|  | | | | |
| Knees | Tender or swollen | 2 | 5 | 7 |
|  | Non tender or swollen | 11 | 14 | 25 |
|  | Total | 13 | 19 | 32 |
|  | | | | |
| Ankles | Tender or swollen | 2 | 6 | 8 |
|  | Non tender or swollen | 7 | 17 | 24 |
|  | Total | 9 | 23 | 32 |
|  | | | | |
| MTP 1 joints | Tender or swollen | 1 | 3 | 4 |
|  | Non tender or swollen | 7 | 21 | 28 |
|  | Total | 8 | 24 | 32 |
|  | | | | |
| MTP 2 – 5 joints | Tender or swollen | 0 | 20 | 20 |
|  | Non tender or swollen | 5 | 103 | 108 |
|  | Total | 5 | 123 | 128 |

*Abbreviations: MCP: metacarpophalangeal, PIP: proximal interphalangeal, MTP: metatarsophalangeal*

Supplementary Table 2. Detailed comparison between PET enhancement and clinical data per entheses site

| **Enthesis** |  | **Visual PET-positive** | **Visual PET-negative** | **Total** |
| --- | --- | --- | --- | --- |
| Supraspinatus insertion | Clinical enthesitis | 2 | 13 | 15 |
|  | No clinical enthesitis | 1 | 16 | 17 |
|  | Total | 3 | 29 | 32 |
|  | | | | |
| Lateral epicondyle humerus | Clinical enthesitis | 3 | 10 | 13 |
|  | No clinical enthesitis | 2 | 17 | 18 |
|  | Total | 5 | 27 | 32 |
|  | | | | |
| Medial epicondyle humerus | Clinical enthesitis | 0 | 7 | 7 |
|  | No clinical enthesitis | 3 | 22 | 25 |
|  | Total | 3 | 29 | 32 |
|  | | | | |
| Trochanter major | Clinical enthesitis | 0 | 8 | 8 |
|  | No clinical enthesitis | 2 | 22 | 24 |
|  | Total | 2 | 30 | 32 |
|  | | | | |
| Quadriceps insertion | Clinical enthesitis | 2 | 2 | 4 |
|  | No clinical enthesitis | 8 | 20 | 28 |
|  | Total | 10 | 22 | 32 |
|  | | | | |
| Patella tendon insertion | Clinical enthesitis | 2 | 6 | 8 |
|  | No clinical enthesitis | 9 | 15 | 24 |
|  | Total | 11 | 21 | 32 |
|  | | | | |
| Achilles tendon insertion | Clinical enthesitis | 3 | 8 | 11 |
|  | No clinical enthesitis | 2 | 19 | 21 |
|  | Total | 5 | 27 | 32 |
|  | | | | |
| Fascia plantaris | Clinical enthesitis | 1 | 1 | 2 |
|  | No clinical enthesitis | 4 | 26 | 30 |
|  | Total | 5 | 27 | 32 |
|  | | | | |
| 1st costochondral joint | Clinical enthesitis | 0 | 16 | 16 |
|  | No clinical enthesitis | 0 | 16 | 16 |
|  | Total | 0 | 32 | 32 |
|  | | | | |
| 7th costochondral joint | Clinical enthesitis | 0 | 9 | 9 |
|  | No clinical enthesitis | 0 | 23 | 23 |
|  | Total | 0 | 32 | 32 |
|  | | | | |
| Spina iliaca posterior superior | Clinical enthesitis | 0 | 13 | 13 |
|  | No clinical enthesitis | 0 | 19 | 19 |
|  | Total | 0 | 32 | 32 |
|  | | | | |
| Spina iliaca anterior posterior | Clinical enthesitis | 0 | 8 | 8 |
|  | No clinical enthesitis | 0 | 24 | 24 |
|  | Total | 0 | 32 | 32 |
|  | | | | |
| Crista iliaca | Clinical enthesitis | 0 | 7 | 7 |
|  | No clinical enthesitis | 0 | 25 | 25 |
|  | Total | 0 | 32 | 32 |
|  | | | | |
| Processus spinosus L5 | Clinical enthesitis | 0 | 3 | 3 |
|  | No clinical enthesitis | 0 | 29 | 29 |
|  | Total | 0 | 32 | 32 |

Supplementary Table 3. Overview of visual PET-positive axial lesions

| **Spine location** | **Frequency of visual PET-positive, n (% of total)** | **Likely PsA related, n (% of total lesions at that location)** | **Likely degenerative related, n (% of total lesions at that location)** |
| --- | --- | --- | --- |
| Processus spinosus | 0 (0) | 0 (0) | 0 (0) |
| Costovertebral joint | 1 (2.0) | 1 (100) | 0 (0) |
| Facet joint | 23 (45.1) | 4 (17.4) | 19 (82.6) |
| Anterior side vertebra | 14 (27.4) | 3 (21.4) | 11 (78.6) |
| Posterior side vertebra | 1 (2.0) | 0 (0) | 1 (100) |
| Inferior endplate vertebra | 1 (2.0) | 1 (100) | 0 (0) |
| Superior endplate vertebra | 1 (2.0) | 0 (0) | 1 (100) |
| Other | 8 (15.6) | 0 (0) | 8 (100) |
| Sacro-iliac joint | 2 (3.9) | 2 (100) | 0 |
| **Total** | **51 (100)** | **11 (21.6)** | **40 (78.4)** |
